# Cancer inequalities in incidence and mortality in the State of São Paulo, Brazil 2001–17

**DOI:** 10.1002/cam4.6259

**Published:** 2023-06-22

**Authors:** Adeylson Guimarães Ribeiro, Jacques Ferlay, Salvatore Vaccarella, Maria do Rosário Dias de Oliveira Latorre, José Humberto Tavares Guerreiro Fregnani, Freddie Bray

**Affiliations:** ^1^ Cancer Surveillance Branch International Agency for Research on Cancer Lyon France; ^2^ Educational and Research Institute Barretos Cancer Hospital Barretos Brazil; ^3^ School of Public Health University of São Paulo São Paulo Brazil; ^4^ A. C. Camargo Cancer Center São Paulo Brazil

**Keywords:** cancer, epidemiology, incidence, mortality, socioeconomic status

## Abstract

**Background:**

Cancer disparities exist between and within countries; we sought to compare cancer‐specific incidence and mortality according to area‐level socioeconomic status (SES) in the State of São Paulo, Brazil.

**Methods:**

Cancer cases diagnosed 2003–2017 in the Barretos region and 2001–2015 in the municipality of São Paulo were obtained from the respective cancer registries. Corresponding cancer deaths were obtained from a Brazilian public government database. Age‐standardized rates for all cancer combined and the six most common cancers were calculated by SES quartiles.

**Results:**

There were 14,628 cancer cases and 7513 cancer deaths in Barretos, and 472,712 corresponding cases and 194,705 deaths in São Paulo. A clear SES‐cancer gradient was seen in São Paulo, with rates varying from 188.4 to 333.1 in low to high SES areas, respectively. There was a lesser social gradient for mortality, with rates in low to high SES areas ranging from 86.4 to 98.0 in Barretos, and from 99.2 to 100.1 in São Paulo. The magnitude of the incidence rates rose markedly with increasing SES in São Paulo city for colorectal, lung, female breast, and prostate cancer. Conversely, both cervical cancer incidence and mortality rose with lower levels of SES in both regions.

**Conclusions:**

A clear SES association was seen for cancers of the prostate, female breast, colorectum, and lung for São Paulo. This study offers a better understanding of the cancer incidence and mortality profile according to SES within a highly populated Brazilian state.

## Graphical abstract

We noted marked increases in incidence rates with increasing SES in the municipality of São Paulo for cancers of the colorectum, lung, female breast, and prostate. For female breast, lung, and to some extent colorectal cancer, both incidence and mortality rates uniformly rose with increasing SES, whereas for cervical cancer, both incidence and mortality rates declined with increasing SES. For prostate cancer, there was a marked increase in incidence rates with increasing SES, though the inverse was seen for mortality.

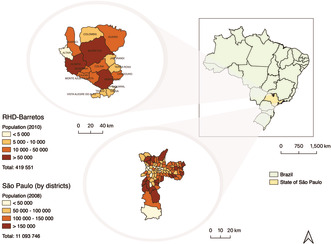

## 1 INTRODUCTION

Cancer is a major public health problem and ranks as the first or second leading cause of premature death in most countries worldwide.^1^ Over 19 million new cases and 10 million cancer deaths were estimated globally in 2020,^2^ with the national scale and profile of cancer clearly linked to corresponding levels of the Human Development Index (HDI).^3^ The Brazilian National Cancer Institute (INCA) estimated that 704,000 new cases of cancer will occur annually during the 2023–2025 triennium, noting differences related to subnational levels of HDI. The southeast region, with the highest HDI, observed age‐adjusted incidence rates 25% higher than the north region, with the lowest HDI.^4^ In terms of cancer mortality, rates in southeast Brazil were 32% and 18% higher in men and women, respectively, compared with the northern region over the period 2001–2020.^5^

Such variations may in part be explained by an increasing exposure to key risk factors already highly prevalent in transitioned countries. It has been consistently estimated that 30%–40% of cancers are attributable to modifiable risk factors that include tobacco smoking, alcohol consumption, unhealthy diet, overweight and obesity, physical inactivity, ultraviolet radiation, and infectious agents.^6^, ^7^, ^8^ Socioeconomic disparities may account for divergent patterns of exposure to these underlying causes, and aggravate accessibility and adherence to programs of cancer prevention, screening and treatment at the population level, which in turn affects the magnitude of incidence and mortality rates for a large number of common cancer types.^1^, ^3^

This study examines cancer incidence and mortality in the northeast and southeast of the State of São Paulo, Brazil, areas with considerable demographic diversity, contrasting cancer profiles according to socioeconomic status (SES) in the Barretos region and the municipality of São Paulo, Brazil. Our study aims to provide insights into how socioeconomic drivers impact on cancer rates, thus informing a more tailored implementation of preventive and curative measures aimed at reducing the disease burden in the region in the coming years.

## 2 METHODS

### 2.1 Study area and population

**FIGURE 1:**
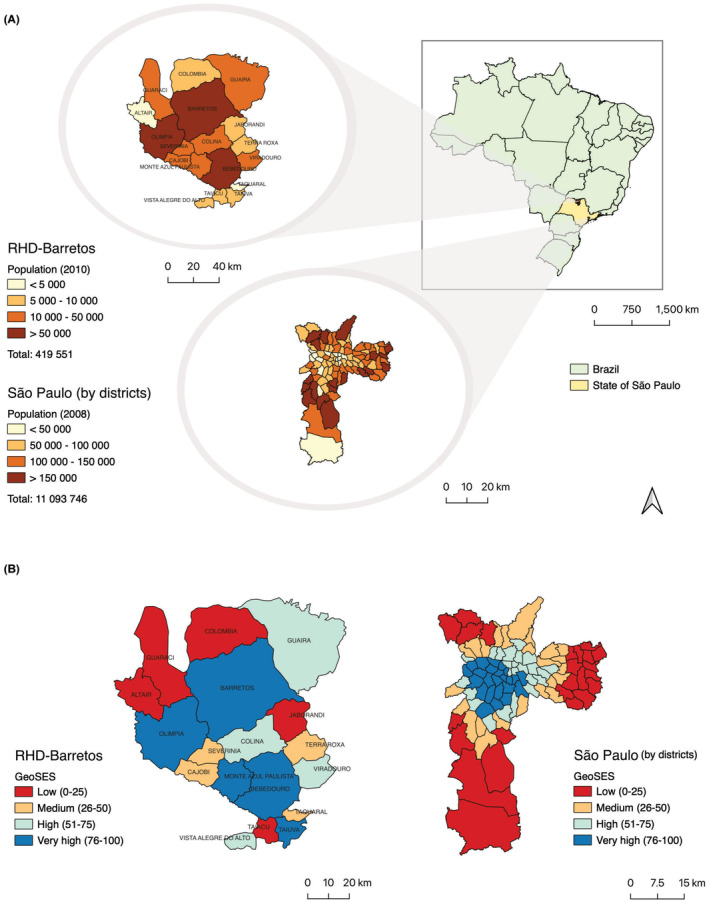
(A) Geographic location and estimated population, and (B) geographic index of the socioeconomic context (GeoSES), in the Regional Health Department (RHD) of Barretos and municipality of São Paulo, Brazil.

This study was conducted in the municipalities belonging to the Regional Health Department of Barretos (RHD) and in the municipality of São Paulo that comprises 96 districts (Figure 1A). The RHD of Barretos is in the northeast of the State of São Paulo and comprises 18 municipalities (Altair, Barretos, Bebedouro, Cajobi, Colina, Colômbia, Guaíra, Guaraci, Jaborandi, Monte Azul Paulista, Olímpia, Severínia, Taiaçu, Taiúva, Taquaral, Terra Roxa, Viradouro, and Vista Alegre do Alto). Collectively, the municipalities occupy an area of 6118.72 km^2^ containing an estimated 445,216 inhabitants in the year 2021.^9^ The municipality of São Paulo, the Capital of the State of São Paulo is located in the southeast of the state covering an area of 1521.202 km^2^ with an estimated 12,396,372 inhabitants in the year 2021.^9^ The two regions offer different demographic profiles, with the RHD of Barretos containing small and medium‐sized cities, whereas São Paulo is a metropolis, being the largest and most populous city in Brazil.

### 2.2 Cancer and population data sources

The number of new cancer cases were extracted for a 15‐year period from the population‐based cancer registries (PBCR) of Barretos (diagnoses 2003–2017) and São Paulo (2001–2015). We considered all new cancer cases based on the International Statistical Classification of Diseases and Related Health Problems 10th Revision (ICD‐10), excluding nonmelanoma skin cancers (NMSC) (C44). Corresponding cancer deaths for the same types were obtained from a complete Brazilian public government database entitled the *Information System on Mortality*, which is part of the Informatics Department of the Unified Health System (DATASUS).^10^ The deaths were acquired by municipalities for the RHD of Barretos and by districts for São Paulo. Data were extracted for all cancers combined (ICD‐10 C00–C97, excluding C44) and six common types in the State: stomach (C16), colorectal (C18–20), lung (C33–C34, including trachea and bronchus), female breast (C50), cervix uteri (C53), and prostate (C61). In terms of overall data quality, both databases were considered as reasonably complete and accurate, on the basis of a comparison of quality indicators of completeness and validity checked with reference cancer registries in the region.^11^

Population data were available by sex and age for the mid‐period (2010), obtained from the DATASUS^10^ for the RHD of Barretos, and to the year 2008 from the Municipal Health Department of São Paulo,^12^ by district, as estimated by the State Data Analysis System Foundation (SEADE) (Figure 1A).

### 2.3 Socioeconomic index

The Geographic Index of the Socioeconomic Context for Health and Social Studies (GeoSES), established using data from the 2010 Brazilian Demographic Census, was used as an indicator of SES. GeoSES is derived from a principal component analysis of seven socioeconomic dimensions: education, poverty, wealth, income, segregation, mobility, and access to resources and services. A detailed description of the GeoSES has been described elsewhere.^13^ We obtained the database GeoSES for the municipalities that comprise the RHD of Barretos (one index by municipality) and the municipality of São Paulo (multiple indexes according to small areas). To investigate the role of SES on cancer incidence and mortality, the GeoSES was stratified into four categories: low (quartile ≤25), medium (quartile 26–50), high (quartile 51–75), and very high (quartile >75) (Figure 1B). As well as differing population characteristics, the two regions vary in socioeconomic profiles, with São Paulo presenting a higher SES on average, but with wide variability in SES across 96 districts, than between the municipalities in the RHD of Barretos.

### 2.4 Statistical analysis

We present age‐standardized incidence or mortality rates (ASR), per 100,000 person‐years based on the 1966 Segi‐Doll World standard population, stratified by cancer GeoSES categories, thus allowing comparisons between populations adjusted for differences in age structure. Using direct standardization, the ASR were estimated from the 10‐year age‐specific rates (ages 0–9, 10–19, …, 60–69, and ≥70 years) by cancer type.^14^ We estimated ASRs by municipality for the RHD of Barretos, and for 96 districts for the municipality of São Paulo. We calculated the Slope Index of Inequality (SII) via linear regression analysis of cancer incidence and mortality rates, taking into account the order of GeoSES categories (most to least deprived area) and the population size for each group. The SII represents the absolute inequality gap across the whole population between the most and least disadvantaged group.^15^, ^16^ The analyses focused on all cancers excluding nonmelanoma and six common types in the State, namely cancers of the stomach, colorectum, lung, female breast, cervix uteri, and prostate. To assess the distribution of cases by districts in São Paulo, we performed a geocoding by residential zip code using the BatchGeo software. Cases of unknown zip code (26.9% of total cases) had district information imputed considering the frequency of each cancer site (C00–C97) across the 96 districts over the 15‐year period, based on those cases that had zip code information. Sensitivity analyses were conducted on the spatial distribution of the ASRs before and after imputation. The distribution of cases in the RHD of Barretos had no missing information related to the municipalities. The cancer‐ and sex‐specific incidence ASR for São Paulo were corrected for cases of unknown age (5.8% of all cancer cases) using an adjustment factor: the total number of cases of cancer divided by the number of cases occurring in individuals of known age.^11^

## 3 RESULTS

The RHD of Barretos has an estimated 419,551 inhabitants in 2010 contrasting with high‐populated São Paulo with an estimated 11,093,746 inhabitants in 2008 (Figure 1A). The population proportions in the lowest and highest SES quartiles had opposing patterns in Barretos and São Paulo. In the RHD of Barretos, higher SES quartiles were seen in municipalities with larger populations (lowest SES quartile: 7.9%; highest SES quartile: 63.9% of total population), whereas, in São Paulo, there was a clear inverse gradient of SES and population size, with socioeconomic levels increasing as population density decreased (lowest SES quartile: 34.1%; highest SES quartile: 15.1% of total population).

**FIGURE 2:**
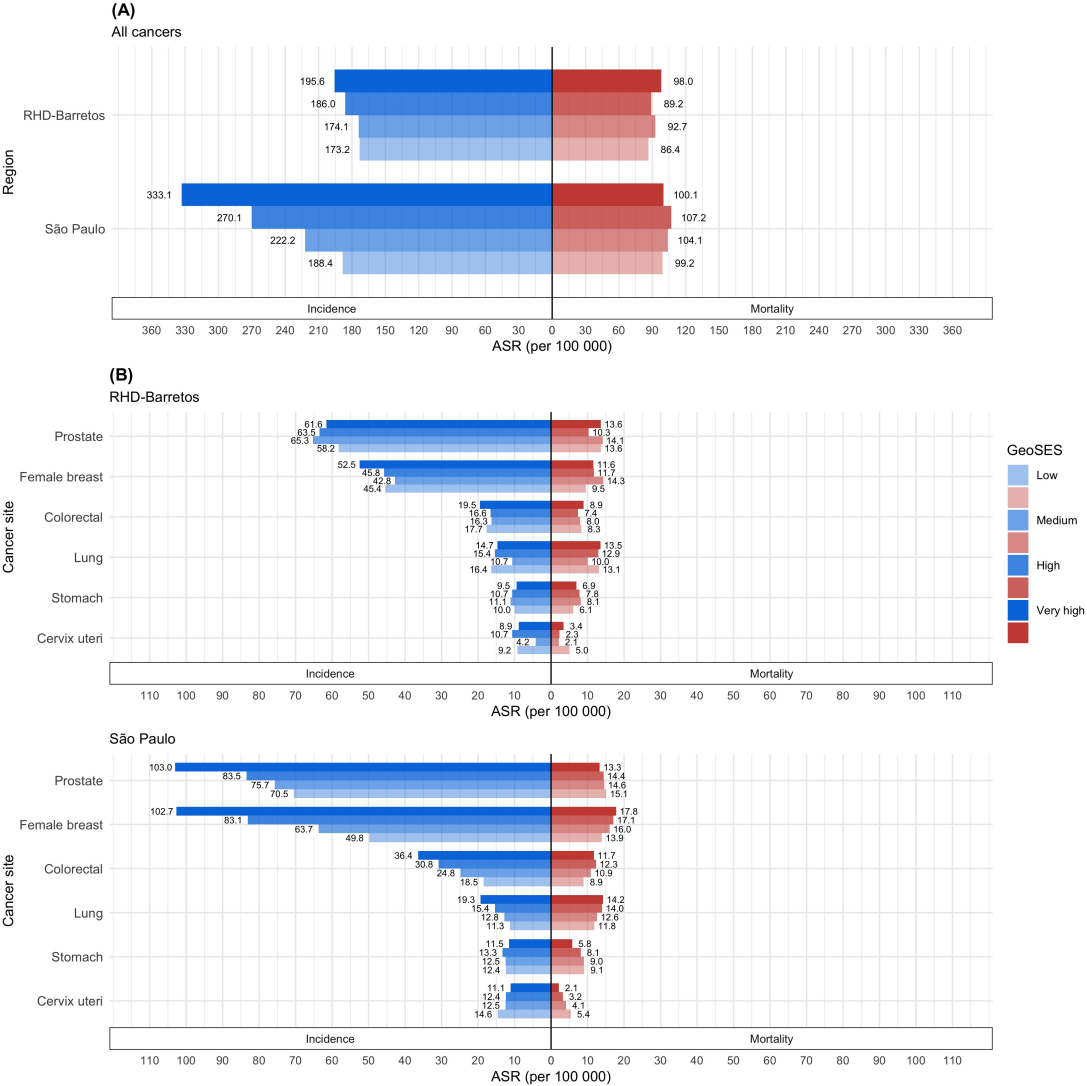
Incidence and mortality age‐standardized rates for (A) all cancers (excluding nonmelanoma skin cancers), and for (B) six common cancers in the Regional Health Department (RHD) of Barretos (2003–2017) and the municipality of São Paulo (2001–2015), both sexes, all ages, by GeoSES in quartiles.

There were 14,628 cancer cases and 7513 cancer deaths in Barretos 2003–2017, and 472,712 corresponding cases and 194,705 deaths in São Paulo 2001–2015 (Table 1). Incidence rates for all cancers combined (excluding nonmelanoma) varied with SES, with the ASR ranging from 173.2 (per 100,000) in low SES to 195.6 in very high SES areas in Barretos, while the gradient was stronger in São Paulo, with rates varying from 188.4 to 333.1 in low to very high SES areas, respectively (Figure 2A). There was less variation in all‐cancer mortality rates, with the ASR in low to very high SES areas ranging from 86.4 to 98.0 in Barretos, and from 99.2 to 100.1 in São Paulo.

**TABLE 1:** New cases, deaths, and age‐standardized incidence and mortality rates (ASR) for lower (L) and higher (H) GeoSES, for all cancers combined and six common cancers, in the Regional Health Department (RHD) of Barretos and municipality of São Paulo, both sexes, all ages.

		RHD‐Barretos (2003–2017)				São Paulo (2001–2015)			
		Incidence		Mortality		Incidence		Mortality	
		New cases	ASR	Deaths	ASR	New cases	ASR	Deaths	ASR
ICD‐10	Cancer site	*N* (% of all sites)	L/H GeoSES	*N* (% of all sites)	L/H GeoSES	*N* (% of all sites)	L/H GeoSES	*N* (% of all sites)	L/H GeoSES
C16	Stomach	792 (5.4)	10.0/9.5	573 (7.6)	6.1/6.9	22,962 (4.9)	12.4/11.5	15,447 (7.9)	9.1/5.8
C18–C20	Colorectal	1445 (9.9)	17.7/19.5	682 (9.1)	8.3/8.9	50,426 (10.7)	18.5/36.4	21,048 (10.8)	8.9/11.7
C33–C34	Lung, bronchus, trachea	1140 (7.8)	16.4/14.7	1023 (13.6)	13.1/13.5	26,279 (5.6)	11.3/19.3	24,486 (12.6)	11.8/14.2
C50	Female breast	2004 (13.7)	45.4/52.5	491 (6.5)	9.5/11.6	74,809 (15.8)	49.8/102.7	17,631 (9.1)	13.9/17.8
C53	Cervix uteri	350 (2.4)	9.2/8.9	130 (1.7)	5.0/3.4	13,262 (2.8)	14.6/11.1	4106 (2.1)	5.4/2.1
C61	Prostate	2211 (15.1)	58.2/61.6	494 (6.6)	13.6/13.6	59,811 (12.7)	70.5/103.0	10,979 (5.6)	15.1/13.3
C00–C97^a^	All cancers	14,628 (100)	173.2/195.6	7513 (100)	86.4/98.0	472,712 (100)	188.4/333.1	194,705 (100)	99.2/100.1

^a^ Excluding nonmelanoma skin cancer (C44).

*Source*: PBCR‐Barretos, PBCR‐São Paulo, Mortality Information System (SIM‐DATASUS).

Figure 2B shows cancer incidence and mortality ASR for prostate, female breast, colorectal, lung, stomach, and cervical cancer according to the four level‐SES in Barretos and São Paulo. These six sites accounted for 54.3% and 45.1% of all cancer cases and deaths in Barretos, with similar proportions (52.5% and 48.1%, respectively) in São Paulo. Slightly higher incidence rates were seen in municipalities with very high versus low SES in Barretos for prostate cancer (61.6 vs. 58.2 per 100,000), female breast (52.5 vs. 45.4), and colorectal (19.5 vs. 17.7) The inverse was seen for lung (14.7 vs. 16.4), stomach (9.5 vs. 10.0), and cervical cancer (8.9 vs. 9.2), with incidence rates somewhat higher in municipalities with low SES.

More striking were the results from São Paulo, with a stepwise gradient in the magnitude of incidence rates clearly observed with increasing SES across the 96 districts for four cancer types. For prostate, female breast, colorectal, and lung cancer, respectively, the incidence ASR was 32% (103.0 vs. 70.5 per 100,000), 52% (102.7 vs. 49.8), 49% (36.4 vs. 18.5), and 41% (19.3 vs. 11.3) higher in districts with very high SES relative to those with low SES. An inverse gradient was in evidence for cervical cancer with higher incidence rates in districts with low (14.6) in comparison to very high SES (11.1). There was little evidence of an SES relation with stomach cancer incidence.

Relative to incidence, the variations in cancer mortality rates with four‐level SES were less marked but nonetheless a mortality‐SES gradient was apparent, at least in São Paulo; the RHD of Barretos presented little variation in the rates for cancer incidence mortality by SES. In the State capital, as with incidence, mortality rates increased in magnitude with increasing levels of SES for both female breast (17.8 vs. 13.9 per 100,000 in very high vs. low districts, respectively), lung (14.2 vs. 11.8), and, less unequivocally, for colorectal cancer (11.7 vs. 8.9). An inverse relation for prostate cancer was observed, with the mortality ASR decreasing as levels of SES rose (13.3 vs. 15.1 per 100,000 in very high vs. low, respectively), the inverse relation to that of incidence. For cervical cancer, the mortality ASR track those of incidence with higher rates in districts with low (5.4) versus very high SES (2.1).

According to the SII analysis (Table 2), significantly higher incidence rates were seen in Barretos for all cancers combined and female breast cancer for the least versus the most disadvantaged groups (rate differences of 32 and 14 per 100,000, respectively). In São Paulo, incidence rates were markedly higher among the least disadvantaged, relative to the most disadvantaged groups for all cancers (172 per 100,000), female breast (65), prostate (34), and colorectal cancer (22), in addition to moderate but significant differences in female breast and lung cancer mortality rates. For cervical cancer, the inverse was seen, with both incidence and mortality rates higher among the most versus the least disadvantaged groups in São Paulo.

**TABLE 2:** Slope Index of Inequality (SII) for all cancers combined and six common cancers in the Regional Health Department (RHD) of Barretos and municipality of São Paulo, both sexes, all ages.

		RHD‐Barretos (2003–2017)				São Paulo (2001–2015)			
ICD‐10	Cancer site	Incidence		Mortality		Incidence		Mortality	
		SII	95% CI	SII	95% CI	SII	95% CI	SII	95% CI
C16	Stomach	2.1	−1.7, 5.8	0.9	−5.2, 6.9	0.2	−5.5, 5.9	3.4	−3.9, 10.7
C18–C20	Colorectal	−5.0	−11.7, 1.7	−2.0	−6.3, 2.4	−22.7	−30.9, −14.5	−4.5	−9.4, 0.4
C33–C34	Lung, bronchus, trachea	−0.7	−16.8, 15.5	−2.6	−11.7, 6.5	−9.3	−19.9, 1.3	−3.4	−5.5, −1.2
C50	Female breast	−14.6	−24.6, −4.6	0.3	−11.8, 12.4	−65.6	−111.2, −20.1	−5.3	−7.2, −3.4
C53	Cervix uteri	−1.0	−20.5, 18.5	−0.6	−9.5, 8.2	4.3	0.2, 8.4	4.2	2.9, 5.4
C61	Prostate	1.4	−18.4, 21.2	−2.1	−17.2, 13.0	−34.9	−87.9, 18.1	1.9	−0.6, 4.5
C00–C97^a^	All cancers	−32.4	−57.3, −7.5	−16.4	−34.5, 1.6	−172.4	−328.2, −16.8	−5.8	−34.8, 23.2

^a^ Excluding nonmelanoma skin cancer (C44).

*Source*: PBCR‐Barretos, PBCR‐São Paulo, Mortality Information System (SIM‐DATASUS).

## 4 DISCUSSION

This study has shown that all‐cancer incidence rates increase with increasing levels of SES in the RHD of Barretos and the municipality of São Paulo. This gradient is considerably more marked for São Paulo overall and for cancers of the prostate, female breast, colorectum, and lung. There are important variations in the direction of the association when cancer‐specific incidence and mortality are compared with SES levels in the capital; for female breast, lung, and to some extent colorectal cancer, both incidence and mortality rates rise with increasing SES. The inverse occurs for cervical cancer, with both incidence and mortality rates in decline with increasing SES. For prostate cancer, there was a marked increase in incidence rates with increasing SES, though this was not seen for mortality, with rates increasing with decreasing levels of SES.

Below, we reflect mainly on the results in São Paulo given cancer‐specific incidence and mortality rates in the RHD of Barretos, while tending to follow the direction of rates by SES seen in São Paulo, were relatively homogenous across the four level of SES, with no clear gradients observed. One possible explanation for these findings is the lesser extent of cancer inequalities in the Barretos region. The region has small‐ and medium‐sized cities with relatively small populations, less variability in SES, and greater accessibility to health services, compared to São Paulo. Another contributory factor to the relative homogeneity is the existence of Barretos Cancer Hospital, a reference center for prevention and cancer treatment in Brazil.^17^, ^18^ An evaluation of trends in access to cancer screening, with a focus on mammography, Pap smears and prostate‐specific antigen (PSA) testing, found inequalities in the city of São Paulo from 2003 to 2015, with access to diagnostic tests greater among more educated individuals.^19^

Our results from the capital are in line with global between‐country variations in the magnitude of cancer according to national levels of human development, for which a positive gradient between cancer incidence rates and HDI has been reported, notably for prostate, female breast, colorectal, and lung cancer.^20^ There are however substantial differences in estimated incidence rates within countries, including Brazil, with incidence rates of breast and colorectal cancer two times higher in the southeast region (with the highest HDI), versus the northern region of Brazil (with the lowest). In contrast, the latter region has reported relatively high incidence rates of stomach and cervical cancer, respectively, with rates two times higher than those in the southeast of the country.^4^ The incidence variations by SES in our study may also reflect differences in accessibility to health services where public and private systems coexist, influencing screening practices and cancer diagnosis. In the Brazilian context, women without health insurance had a 14% lower prevalence of mammographic screening and presumably lower breast cancer detection than those insured.^21^ Similarly, men with private health insurance had a higher prevalence of digital rectal examination (63.3%) than those within the public health system (41.6%), potentially creating a differential in early detection of prostate cancer between the two groups.^22^ The increase of incidence with increasing SES may result in opportunistic screening with PSA and the consequential detection of low‐risk cancers among those with greater access to the health system.

The inverse relationship between socioeconomic development and cervical cancer in São Paulo is in line with previous analyses,^23^ and likely reflects the impact of education and income on access and uptake of preventative services. One study reported women with a lower education in Brazil had a 20% increased risk of late‐stage cervical cancer diagnosis,^24^ while mortality rates in the country have decreased with the increase of HDI.^25^ A regional evaluation in Brazil assessing the performance of the Papanicolaou (Pap) test reported that women with higher levels of education, and with private health insurance were more likely to have a test as recommended according the protocol established by the Ministry of Health.^26^ Persistent infection with high‐risk human papillomavirus (HPV) types is necessary for tumor development. Considering the impact of SES on HPV infection, a nationwide multicenter study in Brazil detected no significant difference in the prevalence of HPV (overall and high‐risk genotypes) across social classes.^27^ However, a lower level of education impacted negatively on knowledge regarding persistent HPV as the underlying cause as well as vaccine acceptance, emphasizing the importance of dedicated educational programs adolescents and young adults and their parents in Brazil to increase vaccination rates.^28^, ^29^

Rates of both incidence and mortality rates of female breast cancer increased incrementally with SES in São Paulo, in accordance with a recent meta‐analysis reporting women with a higher educational level had a significantly increased risk of developing breast cancer.^30^ In Europe, an increase of breast cancer mortality was identified in women with higher SES, which was partially explained by reproductive factors.^31^ Breast cancer mortality was however, as with incidence, higher in regions of high and very high SES in São Paulo, implying alongside declines in fertility rates in these subgroups;^32^ mammography screening has had a less than effective impact, given factors related to adherence among women in Brazil include higher education, higher income, and health insurance.^33^ A spatial evaluation in Brazil reported both HDI and the Gini index strongly correlates with mammographic coverage,^34^ while an evaluation of the first 2 years of the program implemented in the RHD of Barretos in 2003 indicated low SES and low education were important barriers to screening uptake.^35^ Patients with advanced stage diagnosis of breast cancer are at higher risk of death and in Brazil, broader determinants include lower education, low density of mammographic equipment, higher indices of social inequality, and low HDI values,^36^ while lower education and referral via the public health system were associated with longer waiting times for treatment post‐diagnosis.^37^, ^38^

Colorectal cancer incidence rates, as with breast cancer, increased with rising SES in São Paulo, as has been observed in Europe, with rates higher among those with a higher SES compared to lower SES position.^39^ From a global perspective, patterns of colorectal cancer incidence correlate with national human development levels, with rates rising rapidly in transitioning countries.^40^ The changing profile of colorectal cancer occurrence in Brazil can in part be explained by increasing patterns of exposure to modifiable risk factors—mainly lifestyle and behavioral factors already common in affluent populations.^3^ However, colorectal cancer mortality rates were also elevated in regions of high and very high SES in São Paulo, contrary to what might be anticipated given other studies, such as the recent assessment in England where a 10% higher 5‐year survival was detected in the least versus the most deprived group.^41^ While screening programs for colorectal cancer have been implemented in recent decades in many high‐income settings, notably in North America and Europe, and continually improved with advances in technologies and evaluation,^42^ in Brazil, no national programs are yet established. In 2015, Barretos Cancer Hospital implemented an organized program based on fecal immunochemical testing (FIT) followed by colonoscopy in cases of positive FIT, assisting mainly the municipalities from the RHD of Barretos.^43^

Intriguingly, while there were large increases in prostate cancer incidence rates with increasing SES (as seen for female breast, colorectal, and lung cancer), the inverse was seen for mortality, with rates incrementally higher with decreasing SES (as seen for cervical and stomach cancer). The incidence pattern probably relates to a greater access and uptake to diagnostic procedures among more versus less affluent populations in São Paulo city. An evaluation of prostate cancer screening in Brazil showed that the highest prostate cancer incidence was seen in the most affluent regions of the country, an observation positively correlated with levels of life expectancy, greater availability of diagnostic methods, and higher levels of overdiagnosis on account of screening policies.^44^ Similarly, a greater volume of prostate cancer diagnoses was seen among males living in high‐income neighborhoods in Canada,^45^ while incidence rates for distant‐stage prostate cancer increased with increasing poverty levels in the United States.^46^ Indeed, while global differences in prostate cancer mortality between countries indexed with high/very high HDI versus low/medium HDI tend to be rather small,^2^ the slightly higher mortality rates among the least affluent in São Paulo seen in this study are in line with several studies. A US national study reported that lower SES was associated with a higher odds of presenting metastases at the time of diagnosis,^47^ while a Swedish study showed that men with a higher income were less likely to present with advanced prostate cancer and more likely to receive effective treatment, leading to slightly lower mortality rates after 6 years follow‐up.^48^ However, our study did not have access to data on prostate cancer stage according to SES.

Stomach cancer incidence rates did not exhibit large differences in rates by SES with rates two times higher than the INCA estimates for the southeast region of Brazil in 2023.^4^ Mortality rates in São Paulo were however 36% higher in areas with the lowest versus the highest SES, in line with national mortality patterns in Brazil, with stomach cancer mortality rates 17% higher in the northern relative to the southeast region of the country.^5^

While lifestyle and behavioral factors common in affluent populations relate to the socioeconomic determinants of cancer incidence, particularly for breast and colorectal cancer, better access to health care, including cancer screening, time from diagnosis and treatment, and effective treatment options, should increase cancer survival in these populations. An assessment of socioeconomic disparities in breast cancer survival in two Brazilian state capitals found age‐standardized net survival at 5 years of 55.7% for the most deprived and 67.2% for the most affluent women.^49^ Breast cancer patients with private health‐care coverage in Brazil have been shown to have better outcomes for overall survival relative to those in the public system, additionally when stratified by the time from initial diagnosis and from the time of the first recurrence of breast cancer to death.^50^ Delays and barriers to starting treatment for colorectal cancer have been identified in underprivileged regions of Brazil,^51^ and may link to the lower survival proportions observed in the less developed region of the North of the country, compared to the more developed regions of the Southeast and South.^52^

The major strengths associated with our study include the use of a high‐quality incidence database from the PBCR of Barretos and São Paulo, as well as a comprehensive mortality database spanning over 15 consecutive years. Another strong point is the use of a spatial approach and geoprocessing tools that were applied to identify location‐based patterns in the cancer‐specific incidence and mortality rates according to categories of socioeconomic development. The main limitation was the assumption that at the municipal or district level, rates across geographic areas and levels of socioeconomic development were homogenous, given that an exploration of differences in SES existing within more granular geographic units was not possible in this study. Another concern is the need to use imputation methods on over one quarter (26.9%) of the cases in São Paulo where zip code information was missing. A possible final caveat was the limited information available on the population to inform the denominator of the rates. However, there appear to have been no large migration processes or other demographic changes during the study period, and the use of mid‐period population minimizes any changes over the 15‐year study period.

## 5 CONCLUSIONS

While all‐cancer incidence rates increased with incremental levels of SES in both the RHD of Barretos and the municipality of São Paulo, a gradient, as seen for cancers of the prostate, female breast, colorectum, and lung was highly evident in São Paulo. Such findings contribute to a better understanding of the profile of the cancer incidence and mortality within a country and the direct impact of SES on the burden and suffering at the community level. Such results indicate there is an undercurrent of cancer inequities within Brazil that can be explained by differentials in the uptake of preventive, early diagnosis, screening, and curative services within the current health system at the state level.

## AUTHOR CONTRIBUTIONS

**Adeylson Guimarães Ribeiro:** Conceptualization (lead); formal analysis (lead); funding acquisition (lead); investigation (lead); methodology (lead); resources (lead); visualization (lead); writing – original draft (lead); writing – review and editing (lead). **Jacques Ferlay:** Methodology (equal); validation (equal); writing – review and editing (equal). **Salvatore Vaccarella:** Writing – review and editing (supporting). **Maria do Rosário Dias de Oliveira Latorre:** Resources (equal); writing – review and editing (supporting). **José Humberto Tavares Guerreiro Fregnani:** Funding acquisition (lead); project administration (lead); writing – review and editing (supporting). **Freddie Bray:** Conceptualization (lead); methodology (lead); project administration (lead); resources (lead); supervision (lead); validation (lead); visualization (lead); writing – review and editing (lead).

## FUNDING INFORMATION

São Paulo Research Foundation–FAPESP (Grants numbers 2017/03787–2 and 2021/10806–9).

## CONFLICT OF INTEREST STATEMENT

The authors declare no potential conflicts of interest.

## DISCLAIMER

Where authors are identified as personnel of the International Agency for Research on Cancer/World Health Organization, the authors alone are responsible for the views expressed in this article and they do not necessarily represent the decisions, policy or views of the International Agency for Research on Cancer /World Health Organization.

## Data Availability

Cancer incidence data that support the findings of our study are available from population‐based cancer registries of Barretos and São Paulo. Cancer mortality data are available from a Brazilian public government database entitled Information System on Mortality, which is part of the Informatics Department of the Unified Health System (DATASUS).
